# Ensuring Transfusion Safety: Screening Blood Donors for Human Parvovirus B19

**DOI:** 10.7759/cureus.67359

**Published:** 2024-08-21

**Authors:** Swati Kumari, Reuben Kuruvilla Thomas, Krishanamoorthy R, Ramya Barani, Padma Srikanth

**Affiliations:** 1 Microbiology, Sri Ramachandra Institute of Higher Education and Research, Chennai, IND; 2 Transfusion Medicine, Sri Ramachandra Institute of Higher Education and Research, Chennai, IND

**Keywords:** screening methodologies, blood and plasma derivatives, b19v transmission, blood transfusion safety, human parvovirus b19 (b19v)

## Abstract

Ensuring the safety of blood and blood products is a vital aspect of healthcare. The potential for transmission of pathogens through blood and blood products makes transfusion safety a significant concern. Despite advancements in testing methodologies, donated blood products still pose a risk for infection transmission. Human parvovirus B19 (B19V) is a small, single-stranded, non-enveloped DNA virus transmissible parenterally by blood transfusion. B19V causes a wide range of clinical manifestations, which is generally harmless in healthy individuals. B19V infection may cause severe complications, such as aplastic crises, as it affects erythrocyte progenitor cells in individuals with increased erythrocyte turnover. Additionally, B19V can be transmitted from pregnant women to their foetus, potentially causing hydrops fetalis and foetal death. The potential for transmission through blood and blood products makes B19V a significant concern for transfusion safety. In response to the growing recognition of B19V's impact on transfusion safety, various international health organisations have introduced guidelines to minimise its transmission through blood and plasma products. However, the implementation of these guidelines varies globally, with some regions, such as India, still lacking formal protocols for B19V monitoring. This review article explores the existing methodologies for screening blood donors for B19V, assesses the associated transfusion risks, and considers the implications for public health and clinical practice. By emphasising advancements in diagnostic techniques and the challenges of their implementation, this article provides a comprehensive overview of efforts to reduce the transmission of B19V through blood transfusions, thereby ensuring safer blood supplies and improved patient outcomes.

## Introduction and background

Overview of transfusion safety

Transfusion-transmitted infections (TTIs) pose a major concern for healthcare professionals. Nearly 119 million blood donations are collected yearly, with India requiring approximately 14.61 million units annually [1,2]. Efforts have been made to prevent TTIs, such as human immunodeficiency virus (HIV)-1 and HIV-2, human T-cell lymphotropic virus (HTLV) I and II, hepatitis C virus (HCV), hepatitis B virus (HBV), and West Nile virus (WNV) [3]. Emerging infections include WNV, *Trypanosoma cruzi*, *Plasmodium* spp., *Babesia* spp., parvovirus B19 (B19V), dengue virus, and prions causing variant Creutzfeldt-Jakob disease. Some agents, such as human herpes virus-8 (HHV-8), *Borrelia*, and potentially avian flu virus, have a viraemic phase but have not been proven to be TTIs [4]. Screening for infectious agents in blood donors is essential to prevent disease transmission through transfusions. Since the beginning of the 21st century, the European Pharmacopoeia, the Plasma Protein Therapeutics Association (PPTA), and the U.S. Food and Drug Administration (FDA) have established guidelines aimed at reducing the risk of B19V transmission through blood and plasma derivatives.

Importance of screening for infectious agents

Blood transfusion safety has significantly improved due to advancements in donor recruitment, blood screening, sensitive testing assays, and proper clinical utilisation [5]. Traditionally, serologic testing formed the basis of screening, but newer methods such as nucleic acid testing (NAT) have further reduced the "window period" [6]. However, NAT has limitations, especially in detecting low-level viremia in blood components, which may evade detection. Despite these limitations, the combination of serological testing and NAT has significantly reduced the risk of viral transmission through blood transfusions [7]. The risk of infectious agents entering the blood supply is dynamic and can change with emerging pathogens or shifts in epidemiology [8].

Human parvovirus B19 and blood products

B19V is a common contaminant in blood and plasma donations. The virus's small size and lack of a lipid envelope make it resistant to most viral inactivation methods, increasing the probability of transmission through blood products [9]. B19V's notable resistance properties make it an effective model for studying newly emergent viruses that can contaminate blood products. Limiting the B19V viral load in source plasma pools is crucial to minimise transmission risk [10,11].

## Review

Human parvovirus B19 - an overview

Virology and Structure

B19V, belonging to the Parvoviridae family and *Erythrovirus* genus, was first identified by Cossart et al. in 1975 [12]. It is one of the smallest DNA viruses, with virions measuring 18-26 nm [13]. B19V is non-enveloped, with a single-stranded DNA genome of about 5,600 nucleotides [14]. The icosahedral capsid comprises two structural proteins, VP1 and VP2, with VP2 making up 95% of the capsid [15]. VP1 includes the VP1 unique region (VP1u), which is essential for virus entry and the infection outcome [16]. The genome also encodes the non-structural protein NS1, which is crucial for viral DNA replication and inducing apoptosis in host cells [17]. B19V is divided into three genotypes (1, 2, and 3), each with over 10% DNA sequence variation [18]. Despite genomic differences, these genotypes share common biological traits and pathogenic capabilities [19].

Epidemiology

B19V is a globally prevalent pathogen with a seroprevalence rate that varies with age, ranging from 2% to 70%. It causes various clinical manifestations, with an attributable risk of erythema infectiosum (fifth disease) being 100% in children, aplastic crisis 70-80% in individuals with haemolytic disorders, hydrops fetalis 10-15% in pregnant women, and chronic anaemia 50% in immunocompromised patients [20]. Epidemiological studies show significant variations in B19V seroprevalence across regions and age groups. Filatova et al. reported two to three times higher incidence rates (10-20 cases per 100,000 population) typically observed from late winter to early summer in temperate climates, but in tropical climates, the virus is generally present year-round [21]. Broliden et al. noted that epidemic cycles occur every four to five years in temperate climates, necessitating adaptive screening and intervention strategies [22]. Positive cases are spread throughout the year in tropical areas, suggesting sporadic occurrences. However, undetected local outbreaks cannot be ruled out [23]. Additional studies, such as those by Abdelrahman et al. in Qatar and Chirambo-Kalolekesha et al. in Zambia, highlight significant regional variations in B19V prevalence and the need for localised public health strategies [24,25]. Continuous regional surveillance is crucial to effectively adapt public health responses to local epidemiological patterns.

B19V seroprevalence varies significantly with age: approximately 2-21% in children aged one to five years, 30-40% in adolescents, 40-60% in adults, and over 85% in elderly populations [26]. Specific IgG antibodies indicate long-term immunity following infection [20].

Mode of Transmission

**Figure 1:**
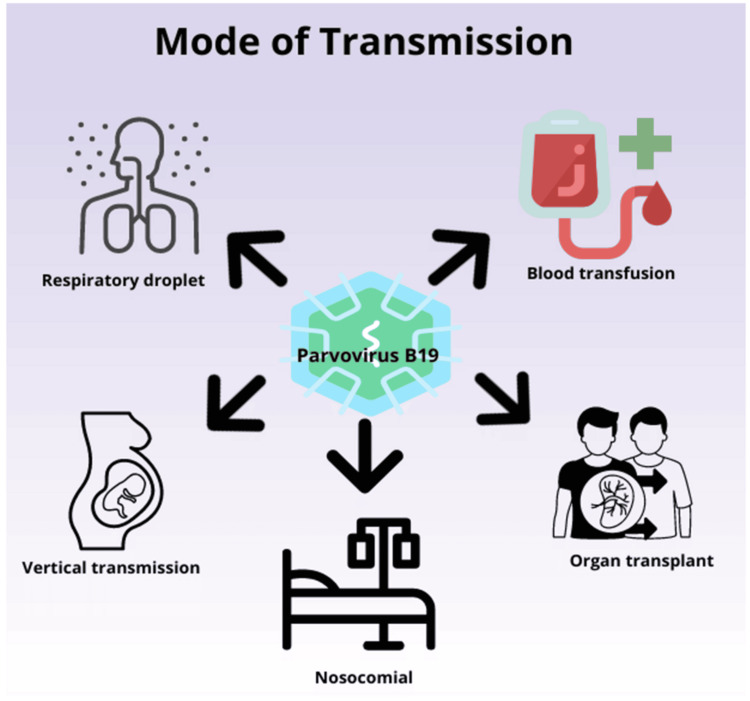
Mode of transmission of parvovirus B19 Figure created by the authors

B19V is primarily spread through droplets from respiratory secretions (Figure 1), with the highest infectivity during the viraemic phase [27]. Common transmission settings include households, daycares, and schools. The virus can also be transmitted vertically and through blood transfusions and organ or bone marrow transplants [28,29]. B19V transmission through blood products is feasible due to the high-level viremia often resulting from primary infections. Asymptomatic individuals in the initial phase of acute infection can have viral loads exceeding 10¹² IU/mL, increasing the risk of iatrogenic transmission [28]. Nosocomial transmission has been documented, such as the detection of B19V in a kidney transplant unit in China, raising concerns for vulnerable immunocompromised populations. Stringent hand hygiene measures and environmental disinfection effectively prevent nosocomial transmission [30].

Pathogenesis and Clinical Manifestations

B19V infection complicates efforts to ensure blood transfusion safety. Two critical factors influence the virus's entry into host cells: the presence of a specific receptor on the host cell surface and a structural component of the virus. B19V specifically targets cells expressing the P blood group antigen (globoside-4 or Gb4) found on erythroblasts, megakaryoblasts, endothelial cells, and foetal myocardial cells. The VP1 unique region (VP1u) is necessary for the virus to enter the host cell. Antibodies against the VP1 component are protective and crucial in clearing the virus. The presence of IgG antibodies confers protection against new infections with any genotype of the virus [16].

B19V viremia begins approximately one week after exposure, peaks within the first two days, and typically lasts about five days. During acute infection, viral titres in peripheral blood can reach up to 10¹⁴ IU/mL [31]. IgM antibodies against B19V appear late in the viraemic stage, around 10-14 days post-infection, and can persist for up to five months, with some patients experiencing longer durations. Specific IgG antibodies become detectable around 15 days after infection, maintaining high levels for several months and persisting long term [32]. Antibodies against the VP1 component of the virus actually determine the fate of B19V infection, as these are protective and play a crucial role in clearing the virus [33]. The presence of IgG antibodies confers protection against any new infection with any genotype of the virus.

Many individuals infected with B19V show no symptoms, with roughly 25% of adults and 50% of children remaining asymptomatic during an outbreak. Symptomatic individuals typically experience mild, non-specific symptoms resembling those of a common cold [34]. The most prevalent clinical manifestation in children is erythema infectiosum, characterised by a distinctive "slapped-cheek" facial rash [35].

The severity of B19V anaemia depends on the host's haematological and immunological condition. B19V infection can lead to severe clinical outcomes in three groups: individuals with conditions that reduce RBC lifespan, such as sickle cell anaemia, thalassemia, or other hemoglobinopathies; immunosuppressed individuals; and pregnant women. B19V infection can cause life-threatening transient aplastic crises in individuals with haematological disorders, chronic anaemia in immunocompromised patients, and hydrops fetalis in pregnant women. For immunocompromised individuals, the inability to produce effective neutralising antibodies may lead to chronic anaemia. The infection can also cause a slight decrease in haemoglobin or haematocrit due to extensive apoptosis of infected erythroid precursor cells, leading to anaemia. However, this anaemia is usually short-lived in most healthy individuals, as the typical lifespan of RBCs is around 120 days. Various studies provided insights into managing chronic anaemia and other severe outcomes, emphasising the critical role of early detection and intervention, particularly in high-risk groups (Table 1) [20,26,35-51].

**Table 1:** Pathogenesis and clinical manifestations of parvovirus B19 infection

Host	Organ affected	Classical disease	Tissue affected by B19V	Pathology known	Management
Immunocompetent					
1. Children	Skin	Erythema infectiosum	Skin and subcutaneous tissue	Immune complex deposition	Saline baths or calamine lotion, antipyretics
2. Adult women	Multiple small joint	Arthropathy	Synovial fluid	Immune complex deposition	Antipyretics non-steroidal anti-inflammatory
3. Pregnant women	Placenta	Hydrops fetalis Recurrent abortion Congenital anemia	Placental tissue	Placental endothelium infection, Erythroblast of fetal liver	Intrauterine blood transfusion, Intravenous and intraperitoneal immunoglobulin
Other symptoms in immunocompetent	Heart	Myocarditis	Myocardial cells	Intracardiac arteriole endothelial cell inflammation	Intravenous immunoglobulin
	Kidney	Focal segmental glomerulosclerosis	Glomerular disease	Immune complex deposition	Intravenous immunoglobulin
	Liver	Acute hepatitis	Hepatocyte	Hepatocyte infection	
	Brain	Meningitis Encephalitis	Central nervous system	Acute cerebellitis Molecular mimicry & autoimmunity to myelin protein	Intravenous immunoglobulin
Individuals with increased red cell turnover	Bone marrow	Severe anemia, Transient aplastic crisis	Erythroid progenitor cell	Erythroid progenitor cell lysis	Blood transfusion Intravenous immunoglobulin
Immunocompromised	Bone marrow	Chronic pure red cell aplasia	Erythroid progenitor cell	Cell cycle arrest	Intravenous immunoglobulin, Empirical plasmapheresis

During pregnancy, B19V infection can result in abortion or non-immune hydrops fetalis [20,37]. In the foetus, the infection can cause myocarditis, heart failure, neurological abnormalities, or congenital anaemia, potentially leading to hydrops fetalis [38]. Additionally, B19V infection is associated with the onset or progression of autoimmune diseases, such as rheumatoid arthritis (RA) and systemic lupus erythematosus (SLE) [39]. B19V infection has also been linked to conditions such as meningitis, encephalopathy, epilepsy, myocarditis, dilated cardiomyopathy, and autoimmune hepatitis [20,39].

B19V Outcome in Blood Donors

Asymptomatic donors can donate blood due to the frequent absence of symptoms in acute infections, especially among adults. When symptoms do occur, adults may experience mild, non-specific signs that resemble a common cold or flu, such as low-grade fever, fatigue, headache, sore throat, malaise, and joint pains (arthralgia) [34].

Haematological course: Researchers in Germany investigated how B19V infection affects blood count levels among blood donors. The study compared 345 samples containing detectable B19V DNA to 100 samples without B19V DNA. The findings revealed no differences between the two groups in leukocyte, erythrocyte, or platelet counts. However, B19V DNA-positive samples had significantly lower mean haemoglobin, haematocrit, mean corpuscular volume (MCV), and mean haemoglobin concentration (MCHC) compared to B19V DNA-negative controls [52].

Immunological course: Anderson et al. detailed the typical antibody response in experimentally B19V-infected individuals. They observed that anti-B19V IgM antibodies appeared during the second week of infection, followed by the emergence of anti-B19V IgG antibodies. However, they did not specify the targeted epitopes, such as NS1, VP1, or VP2 [31]. According to the study by Emmanuel et al., viral epitopes of B19V include specific regions on the VP1 and VP2 capsid proteins. These epitopes are essential for inducing immune responses, making them crucial targets for developing vaccines and diagnostic assays. The VP1 unique region (VP1u) is particularly significant, playing a key role in eliciting protective antibodies essential for the immune system's defence against the virus [53].

A pioneering study by researchers from Germany and Austria investigated how the humoral immune response evolves concerning B19V DNA levels. This research involved analysing follow-up samples from 50 donors infected with B19V. In the first samples gathered during the acute phase of infection, around one-third of the blood donors showed detectable IgG antibodies targeting VP1 and VP2 viral capsid proteins. After 12 weeks, the first follow-up sample showed detectable IgG antibodies in every donor against the viral capsid protein [54].

Implications for Blood Transfusion

Risk of transmission: One significant risk for blood transfusion recipients is the potential transmission of B19V from asymptomatic donors. B19V can be present in high titres in donors' blood who show no symptoms, leading to an unnoticed transmission risk [55].

High viremia: During the acute phase of B19V infection, viral load in the blood can reach very high levels (up to 10¹⁴ IU/mL). Blood products collected during this period significantly increase the risk of TTIs [55,56].

Clinical Implications

Immunocompromised recipients: Immunocompromised individuals, such as those with HIV/acquired immunodeficiency syndrome (AIDS), cancer patients undergoing chemotherapy, or organ transplant recipients, are at a higher risk of developing severe complications from B19V infection. These patients may experience chronic anaemia due to the persistent suppression of erythropoiesis [25].

Hematologic disorders: Recipients with underlying hematologic disorders, such as sickle cell anaemia, thalassemia, or other hemoglobinopathies, are particularly vulnerable. B19V can precipitate a transient aplastic crisis in these patients, leading to severe anaemia requiring urgent medical intervention [36].

Pregnant women: Anaemia (most often mild, sometimes severe) complicating pregnancy is a common condition encountered in antenatal practice. Pregnant women receiving blood transfusions are at risk of transmitting B19V to the foetus, which can cause hydrops fetalis, severe foetal anaemia, and even foetal death. This vertical transmission is a significant concern for maternal and foetal health [57].

Autoimmune and inflammatory conditions: B19V infection has been linked to the onset or exacerbation of autoimmune diseases such as rheumatoid arthritis and systemic lupus erythematosus. Blood transfusion recipients with pre-existing autoimmune conditions may experience worsening symptoms or new-onset autoimmune disorders [58].

Screening and Diagnostic Methods for B19V

Nucleic acid testing (NAT): NAT is a highly sensitive and specific method for detecting B19V DNA in blood and plasma products. It involves the amplification of viral DNA using polymerase chain reaction (PCR) techniques, which are capable of identifying even low levels of viral DNA in donor blood samples. NAT can detect B19V during the viraemic phase of infection when viral loads are the highest, with a sensitivity threshold as low as 10³ IU/mL [59]. Many countries have adopted NAT for screening blood donations, often using mini-pool testing where multiple samples are pooled together for analysis. NAT reduces the risk of TTIs by identifying infected donors before distributing blood products [60]. An interesting aspect of B19V viremia is that it is short-lived, unlike infections with other blood-borne viruses, such as HIV/HBV/HCV, where viremia is persistent.

**Figure 2:**
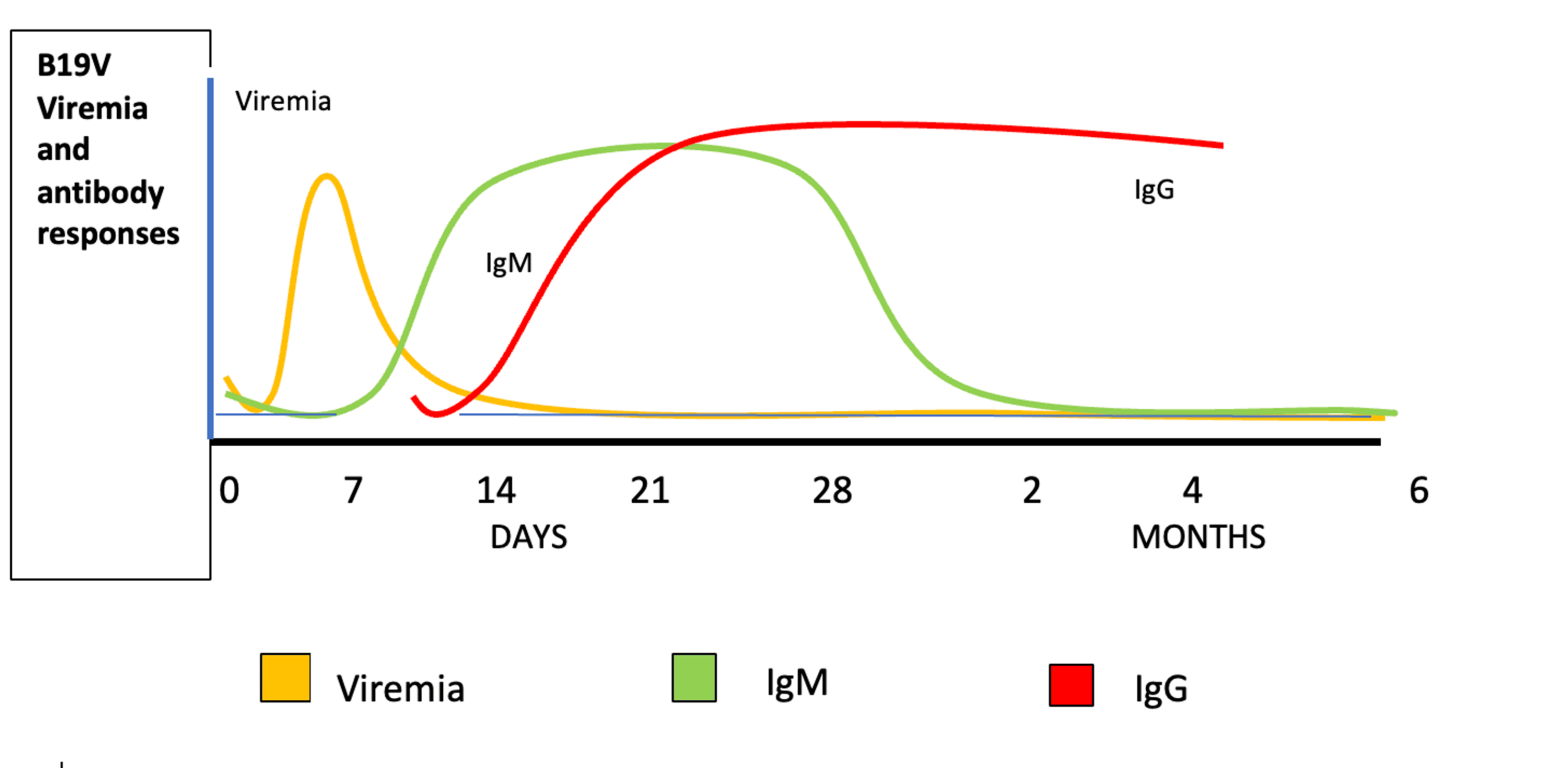
Markers of parvovirus B19 infection Figure created by the authors

Serological testing (IgM, IgG antibodies): Serological testing for B19V involves detecting specific blood antibodies (IgM and IgG), indicating past or current infection. IgM antibodies appear 10-14 days after infection and can persist for several months, indicating a recent or acute B19V infection. IgG antibodies become detectable around 15 days post-infection and remain long term, indicating past exposure and immunity (Figure 2). High levels of IgG antibodies in a donor suggest previous infection and a low risk of a viraemic phase [61]. Serological testing helps identify donors previously exposed to B19V and those currently experiencing an acute infection. Countries may use serological tests alongside NAT to ensure comprehensive screening of blood donors [62].

Comparison of Different Screening Methods: NAT vs. Serological Testing

The evolution of blood transfusion safety has been marked by significant technological advancements, particularly the shift from basic serological testing to more sophisticated NAT. The limitations of serological tests were inadequate in detecting low-level viremia. This gap necessitated the adoption of NAT, significantly reducing the residual risk of TTIs, particularly during the window period of infections.

Additional studies in India (2014) and Germany (2019) emphasise the importance of NAT in reducing the incidence of TTIs and highlight the continuous need for technological advancements [63,64]. The development of multiplex NAT assays has further improved detection sensitivity and allowed for the simultaneous screening of multiple pathogens.

NAT is more sensitive and specific for detecting active B19V infection due to its ability to amplify viral DNA. It is particularly useful during the viraemic phase. Serological tests are less sensitive than NAT but provide valuable information about the immune status of donors through the detection of IgM and IgG antibodies. NAT detects B19V during the viraemic phase, making it effective for preventing TTIs from donors with high viral loads. Serological tests detect recent (IgM) and past (IgG) infections, offering a broader understanding of donor infection history [65]. Combining NAT and serological testing provides the most comprehensive screening approach, capturing active infections and immune status. This dual approach enhances blood safety by minimising the risk of TTIs from B19V [66].

Certain countries, such as Germany, the Netherlands, and Japan, have enacted specific measures to mitigate B19V transmission risk to enhance the safety of blood and blood products [3]. The Japanese Red Cross (JRC), in September 1997, introduced a haemagglutination assay to screen for B19V in all blood donations until 2007. In 2008, the JRC replaced the screening method with a chemiluminescent enzyme immunoassay to enhance the assay's sensitivity. The sensitivity of this test is around 10⁷ IU/mL with positive B19V DNA donor samples [67]. The testing for B19V DNA in blood donors commenced in Germany in 2000. A mini-pool real-time NAT was introduced to facilitate this, allowing for simultaneous testing of up to 96 donations. Blood products with a viral load of ≥10⁵ IU/mL for B19V were subsequently disposed of to maintain safety [54]. The Netherlands Health Council recommended a high-risk group approach for cellular blood products. This involves supplying "B19V-safe" cellular blood products sourced from donors with anti-B19V IgG antibodies identified in two blood samples taken at least six months apart for specific at-risk groups. These groups include pregnant women, individuals with haemolytic disorder, and immunocompromised individuals who also lack detectable B19V-specific antibodies [68]. In Poland, donated blood is universally screened for B19V using NAT-based algorithms [69].

Prevalence of B19V in Blood Donors

Numerous studies have shown significant variation in B19V prevalence among blood donor populations: IgG antibodies range from 6% to 79.1%, IgM antibodies from 0.72% to 7.53%, and B19V DNA from 0% to 1.3% [31,52,61,67,70-76]. While the results of these studies are generally consistent, direct comparison is challenging due to variations in study population sizes, pool sizes, and the use of tests with different sensitivities for B19V DNA detection. The prevalence of B19V DNA can be significantly influenced by the study timing, especially if the study duration does not cover an entire year. Additionally, it is known that the B19V outbreak can occur in specific years, leading to an increased number of acute infections, while other years may have only a few B19V infections [75,76]. Table 2 provides detailed prevalence data from various countries, summarising the prevalence of B19V DNA, IgM, and IgG antibodies in blood donors across different regions [24,25,52,55,61,67,77-104]. It is also noteworthy that approximately 30% of potential blood donors will not have any antibodies against B19V and will, therefore, be at risk of acquiring a new infection.

**Table 2:** Prevalence of B19V reported in blood donors across various countries PCR=Polymerase chain reaction, ELISA=Enzyme‐linked immunosorbent assay Note: Data are represented as the number of positive cases (N) and percentage (%) of the total sample size. Prevalence of B19V reported in blood donors across various countries

Study	Geographical location	Sample size &Type	Method of detection	Prevalence		
				DNA positive (N, %)	Anti-B19V IgM (N, %)	Anti-B19V IgG (N, %)
Filatova et al., 2010 [21]	Russian	1000	Real‐time PCR	10 (1%)	-	-
Abdelrahman et al., 2021 [24]	Qatar	5026 and 930 Blood donor	Real-time PCR, ELISA	70 (1.4%)-	- 20 (2.1%)	- 561 (60.3%)
Chirambo‐Kalolekesha et al., 2018 [25]	Zambia	192	ELISA	-	30 (0.15%)	-
Juhl et al., 2014 [52]	Germany	23,889	Real‐time PCR	157 (0.65%)	-	-
Healy et al., 2023 [55]	Germany	1,67,123 Blood donor	Real-time PCR	22 (0.013%)	-	-
Manaresi et al., 2004 [61]	Italy	446	ELISA	-	-	353 (79.14%)
Sakata et al., 2008 [67]	Japan	1,035,560 and 417	Chemiluminescent enzyme immunoassay, real‐time PCR	101 (0.23%)	-	-
Sun et al., 2023 [77]	China	17,187 Plasma donor	Real-time PCR	44 (0.26%)	-	-
Mengyi et al., 2023 [78]	China	10,720 Blood donor	Real-time PCR	58 (0.14%)	-	-
William et al., 2022 [79]	England	76,065 Blood component, plasma product	Real-time PCR	80 (0.003%)	-	-
Rabab Hasanain et al., 2021 [80]	Egypt	500 Blood donor	Real-time PCR and ELISA	15 (3%)	31 (6.20%)	401 (80.20%)
Slavov et al., 2019 [81]	Brazil	480 Blood donor	Real-time PCR and ELISA	9 (1.87)	-	258 (53.75%)
Francois et al., 2019 [82]	South Africa	1500 Blood donor	Real-time PCR	14 (0.93%)	-	-
Raturi et al., 2018 [83]	India	800 Blood donor	ELISA	-	11 (1.4%)	273 (34.1%)
Goral et al., 2018 [84]	Turkey	988 Blood donor	ELISA	-	39 (3.95%)	582 (58.9%)
Faddy et al., 2018 [85]	Australia	2221 Blood donor	ELISA	-	-	1360 (61.2%)
Osman et al., 2017 [86]	Sudan	110	Nested PCR	8 (7.27%)	-	-
Slavov et al., 2016 [87]	Brazil	91	Real‐time PCR	1 (1.09%)	-	-
Ou et al., 2016 [88]	China	10,452	Real‐time PCR	6 (0.057%)	-	-
Han et al., 2015 [89]	China	5030	Real‐time PCR	3 (0.059)	-	-
Kumar et al., 2013 [90]	India	1633	ELISA	-	123(7.53)	-
Grabarczyk et al., 2012 [91]	Poland	980	Real‐time PCR	1 (0.102%)	-	-
Mahmoodian‐Shooshtari et al., 2011 [92]	Iran	1640	Semi‐nested PCR and ELISA	-	8 (0.48%)	676 (41.21%)
Ke et al., 2011 [93]	China	3957	Real‐time PCR	23 (0.58%)	-	-
Oh et al., 2010 [94]	South Korea	10,032 and 928	Real‐time PCR, ELISA	10 (0.009%) -	- 7 (0.069%)	- 551 (5.49%)
O’Bryan et al., 2010 [95]	USA	282	ELISA	-	-	162 (57.44%)
Johargy, 2009 [96]	Saudi Arabia	578	ELISA	-	-	441 (76.29%)
Mahmoudi et al., 2008 [97]	Iran	730	Semi‐nested PCR and ELISA	0	4 (0.54%)	338 (46.30%)
Kleinman et al., 2007 [98]	USA	5020	Real‐time PCR	44 (0.87%)	-	-
Wei et al., 2006 [99]	China	184	ELISA	-	-	102
Henriques et al., 2005 [100]	Portugal	5025	Real‐time PCR	6 (0.119%)	-	-
Thomas et al., 2003 [101]	Belgium	16,859	Nested PCR	27 (0.160%)	-	-
Muñoz et al., 1998 [102]	Spain	136	ELISA	-	-	88 (64.70%)
Letaïef et al., 1997 [103]	Belgium and Tunisia	819	ELISA	-	11 (1.34%)	572 (69.84%)
Yoto et al., 1995 [104]	Japan	1000	Nested PCR	6 (0.6%)	-	-

Case studies

Japan: Zanella et al. reported a case of TT-B19V infection in a 22-year-old female patient with thalassemia major. She presented with an aplastic crisis, which was followed a week later by transient heart failure and acute tricuspid valve incompetence [105]. Yu et al. discovered that 14 out of 869 (1.6%) recipients were B19V DNA positive and showed seroconversion after receiving red blood cell transfusions, as confirmed by molecular analysis of linked donor and recipient samples [60]. The JRC haemovigilance system gathered clinical reports on potential TT-B19V from 1999 to 2008. Eight patients have been identified with TT-B19V resulting from a blood transfusion. Four patients developed sustained anaemia and pure red blood cell aplasia, while one patient developed pancytopenia [106]. In a study by Yoto et al. on 1,000 serum samples from blood donors, 0.6% tested positive for B19V DNA, a rate significantly higher. Five of these samples also had anti-B19 IgM, indicating acute infection [104].

United Kingdom: Between 1996 and 2016, the UK's Serious Hazards of Transfusion (SHOT) registry documented only a single instance of significant illness resulting from a TT-B19V infection [107].

France: A sickle cell disease recipient developed erythroblastopenia following a red blood cell concentrate transfusion [108].

The reported cases are likely just the "tip of the iceberg," indicating a much larger, often hidden, burden of TT-B19V infections. This emphasizes the need for improved screening, better clinical awareness, and more comprehensive surveillance systems to ensure transfusion safety.

Global regulatory guidelines

**Figure 3:**
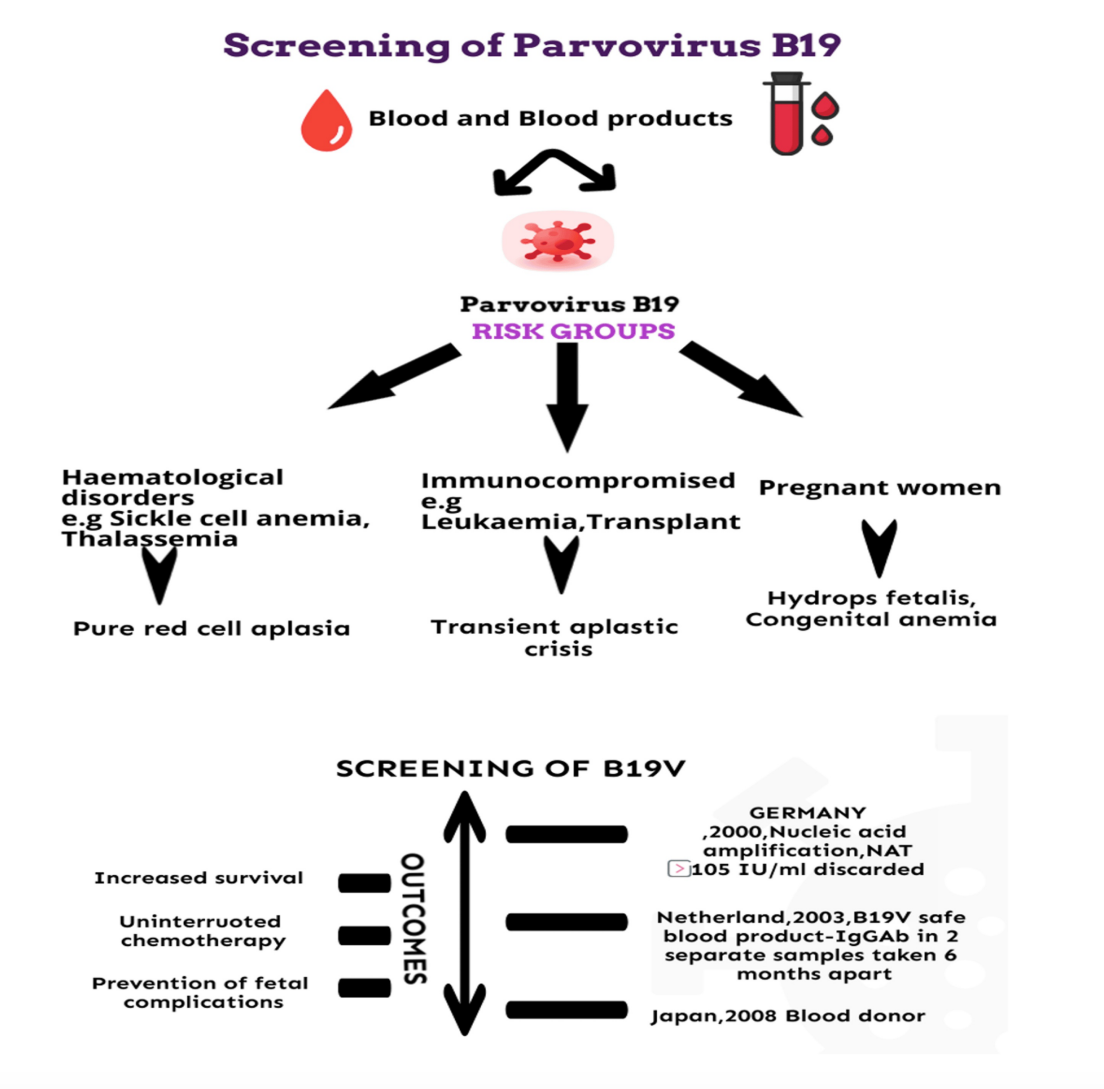
B19V screening algorithm and outcome Figure created by the authors.

Since the early 21st century, various regulatory bodies have established guidelines to mitigate the risk of B19V transmission through blood and plasma products (Figure 3).

European Pharmacopoeia and the Plasma Protein Therapeutics Association (PPTA): These organisations recommend limiting B19V DNA levels to 10⁴ IU/mL in plasma pools used for manufacturing plasma derivatives. These guidelines aim to minimise transmission risk through products such as clotting factors, immunoglobulins, and albumin [109].

U.S. Food and Drug Administration (FDA) guidelines: The FDA has issued similar guidelines, emphasising the importance of limiting B19V DNA levels in plasma pools. Additionally, the FDA requires that NAT assays used for screening detect all three B19V genotypes to ensure comprehensive coverage [110].

The FDA, European Pharmacopoeia, and the PPTA have recommended limiting B19V DNA levels in plasma derivatives to 10⁴ IU/mL [110-113]. While these guidelines provide a framework for reducing B19V transmission risk, implementation varies globally. Some countries, such as India, with many vulnerable populations, currently lack specific documentation or regulations for monitoring B19V, highlighting the need for a more uniform approach to transfusion safety.

The European Centre for Disease Prevention and Control (ECDC) has issued a Threat Assessment Brief on the increased circulation of B19V in the EU/EEA as of June 2024. This report emphasises the need for heightened awareness among public health authorities and Substances of Human Origin (SoHO) professionals, especially for vulnerable populations such as pregnant women, immunosuppressed individuals, and those with chronic haematological conditions. The ECDC recommends enhancing risk communication and considering additional screening measures to manage and mitigate the potential health impacts. The findings underscore the importance of ongoing monitoring and targeted public health interventions [114].

Substantial disparities exist in screening practices for B19V across countries. For example, Japan's early adoption of haemagglutination assays in 1997 and the subsequent shift to chemiluminescent enzyme immunoassays in 2008 reflect a proactive approach to enhancing detection sensitivity and accuracy [67]. In contrast, Germany implemented mini-pool NAT in 2000, exemplifying a robust strategy to maintain high detection standards while managing costs [52]. This underscores the diversity in strategies driven by each country's unique healthcare infrastructure and economic considerations.

A study on Dutch blood donors reveals a complex landscape where economic and infrastructural limitations significantly impact the effectiveness of B19V screening, particularly in low-resource settings [115]. The need for international collaboration and support to bridge the gap in screening capabilities is critical for ensuring comprehensive transfusion safety globally.

Contamination of plasma derivatives

Plasma derivatives, particularly coagulation factors, are at higher risk of B19V contamination due to large volumes of plasma pooling from multiple donors [116]. Documented evidence shows a temporal link between administering plasma products and outcomes such as viremia and seroconversion in recipients. Additionally, studies have shown that groups receiving clotting factors have a significantly higher prevalence of B19V-specific antibodies than control groups [117]. In China, studies have reported B19V DNA contamination rates ranging from 5.45% to 100% in plasma pools, depending on the region and screening procedures used [118,119]. The small size and absence of an envelope make B19V resistant to many viral inactivation methods used in plasma derivative production [103,111].

A study conducted in Atlanta, United States of America, found that individuals receiving plasma-derived medicinal products were 1.7 times more likely to have IgG antibodies to B19V than non-exposed populations [120]. Another study in the United Kingdom revealed that one-fourth of albumin samples and all factor VIII concentrates were affected. One-fifth of intravenous and three-fourths of intramuscular immunoglobulin preparations were also impacted [121]. According to Alter et al., more than 60% of factor VIII, factor IX, and prothrombin complex concentrates, along with plasma pools, were found to contain B19V DNA, with viral loads varying between 1×10 and 1×10⁸ genome equivalent/mL [4].

Strategies for reducing contamination

Since B19V is thermostable and lacks a lipid envelope, it is not destroyed by the chemical and physical treatments used to inactivate lipid-enveloped viruses, such as HIV and hepatitis B and C [39].

Common viral inactivation techniques include heat treatment (e.g., pasteurisation and dry heat treatment), solvent/detergent treatment, ultraviolet (UV) irradiation, gamma irradiation, low pH treatment, and nanofiltration. These methods are designed to disrupt or remove viruses, ensuring that plasma-derived products are safe for therapeutic use. Combining multiple inactivation techniques can provide a robust defence against B19V, thereby enhancing the overall safety of blood products [54,122,123].

Limiting the viral load in plasma pools is essential to mitigate the B19V transmission risk through plasma derivatives. Implementing universal NAT screening for B19V in plasma pools and setting a stringent cut-off level for acceptable viral loads can significantly reduce the risk of contamination. Additionally, enhancing manufacturing processes to include more effective viral inactivation and removal steps can further improve the safety of plasma derivatives.

Challenges and limitations

Despite these measures, screening for B19V presents several challenges. The asymptomatic nature of the infection in many donors complicates the identification of those carrying the virus. Additionally, the persistence of B19V DNA in various tissues poses a risk of reactivation, which is not easily detectable by standard screening methods [124,125].

Persistence and reactivation

One challenge is B19V DNA persistence in various tissues long after acute infection, potentially reactivating under immunosuppressed conditions. Studies have found B19V DNA in tissues such as the liver, heart, and synovia, even in individuals who have cleared the acute infection phase. This persistence complicates efforts to ensure transfusion safety, as reactivation could lead to asymptomatic but infectious donations. Research has demonstrated that B19V DNA can remain in multiple tissues, such as the liver, heart, tonsils, and synovial membranes [126-131]. Post-acute infection, these tissues are proposed to release free B19V DNA, rather than fully formed virions, into the plasma. This persistence is of particular concern in immunocompromised individuals, who may experience reactivation of the virus, leading to potential transmission through blood and plasma products [132].

The persistence and potential reactivation of B19V DNA highlight the need for continuous monitoring and robust screening methods. Current screening techniques primarily detect active infections but may not identify donors with latent or low-level reactivated diseases, posing a risk to recipients.

Despite advancements in screening technologies, significant challenges remain in reducing B19V transmission through blood products. Studies such as those by Azzi et al. and Ke et al. highlight the asymptomatic nature of the infection in many donors, complicating carrier identification [133,93]. Additionally, the persistence of B19V DNA in various tissues poses a reactivation risk not easily detectable by standard screening methods [125]. They highlight significant issues that current screening technologies cannot fully address, emphasising the need for ongoing research and development of more effective detection methods.

Future advancements, such as high-sensitivity NAT assays and Clustered Regularly Interspaced Short Palindromic Repeats (CRISPR)-based diagnostic tools, promise to enhance the efficiency and comprehensiveness of blood screening processes [134]. We believe harmonising international guidelines and standards is essential to ensure consistent blood safety practices globally.

Immune response and antibody protection

Another challenge in the study of blood transfusion safety concerning B19V is that the presence of anti-B19V IgG and IgM antibodies in donors does not always prevent the transmission of the virus to recipients. This indicates that solely relying on antibody detection may be insufficient to ensure the safety of blood products. A more comprehensive approach is required to minimise the risk of TT-B19V infections. This should include NAT and monitoring viral loads to identify and manage cases where antibodies are present but not protective. Additionally, the biphasic nature of B19V viremia, with two distinct peaks, further complicates the detection and prevention of transmission, necessitating ongoing vigilance and advanced screening methods [20].

Other parvovirus and blood-borne transmission

B19V was previously considered the only pathogenic parvovirus causing human infection until the recently discovered human bocaparvoviruses (HBoV1) and parvovirus 4 (PARV4). These newly discovered viruses' epidemiology and disease associations still need to be explored [63]. PARV4, identified in 2005 in an intravenous substance user, has been found in plasma pools and clotting factor concentrates, indicating blood-borne transmission [135]. The prevalence of PARV4 varies geographically, with some regions reporting higher detection rates. HBoV1 was detected in children with respiratory illness in the same year and showed high seroprevalence [136]. However, its detection in blood donors is limited, likely due to low-level viremia. Further studies are needed to explore these newly discovered parvoviruses' clinical significance and transmission routes.

Future perspectives

Advancements in screening technology: Future advancements promise to improve B19V detection in blood donors. Developments in high-sensitivity NAT assays could lead to more sensitive and specific tests for B19V, enabling more reliable detection of low-level viremia [119]. Integrating multiplex testing, which can screen for multiple pathogens, including B19V, could also become standard practice, enhancing the efficiency and comprehensiveness of blood screening processes.

Implementation of universal screening: As technology advances, routine NAT screening for B19V in all blood donations may become more feasible and cost-effective, providing additional safety for transfusion recipients [137].

Improved risk assessment and management: Enhancing risk assessment and management strategies will improve B19V screening programs. Implementing risk-based screening that relies on donor risk profiles and epidemiological data could significantly boost the efficiency and effectiveness of these programs [69]. Moreover, using big data and machine learning to analyse donor and recipient information could help identify patterns and risk factors related to B19V transmission, enabling more informed and precise screening practices.

Global standardization and guidelines: International guidelines and standards for B19V screening in blood donors could ensure a consistent approach across countries, improving global blood safety [110-113].

Public health initiatives: Awareness campaigns to educate healthcare providers and the public about B19V could enhance understanding and improve the management of associated risks. Additionally, ongoing monitoring and surveillance of B19V prevalence in the donor population and the general public are essential. These efforts will help adapt screening strategies to evolving epidemiological trends, ensuring a responsive and practical approach to B19V management.

Vaccination programs: No commercially available vaccine for B19V exists currently. Several potential vaccine candidates are being explored, including recombinant virus-like particles (VLPs), DNA vaccines, and protein subunit vaccines [138]. Developing a B19V vaccine could drastically reduce the prevalence of the virus in the donor population, lowering the necessity for extensive screening measures. Implementing vaccination programs specifically for blood donors could diminish the risk of B19V transmission through blood products.

Economic considerations: A study by van Hoeven et al. showed that the cost-effectiveness of anti-B19V screening is similar to that of other blood safety interventions. This finding emphasises the need for a balanced approach that considers cost-effectiveness, perceived and actual risks, uncertainties, and available alternatives when implementing B19V screening in blood products [115]. The estimated cost for B19V testing in the Indian subcontinent ranges from INR 1,500 to INR 3,000 per NAT test and INR 500 to INR 1,200 per serological test. Universal NAT screening could add ₹18-₹36 billion annually to healthcare costs, while serological testing could cost ₹6-₹14.4 billion annually. To mitigate these financial burdens, pooled testing or targeted testing for high-risk groups is recommended as a more cost-effective approach [83].

## Conclusions

Ensuring the safety of blood transfusions is crucial, especially regarding B19V, due to its prevalence among blood donors and the virus's persistence in asymptomatic individuals. Despite advancements in screening technologies and guidelines from regulatory bodies, the risk of B19V transmission persists. Approximately 30% of potential blood donors lack B19V-specific antibodies, making them susceptible to new infections that often go undetected, allowing the virus to enter the blood supply. NAT and serological testing are essential strategies to identify active and past infections. Countries such as Japan and Germany have shown that robust B19V screening programs effectively reduce transmission risks. However, the absence of specific guidelines in countries such as India highlights a critical gap in global transfusion safety. The most effective prevention of TT-B19V infections includes general blood donor screening for B19V DNA using NAT, antibody testing for anti-B19V IgG, and implementing pathogen reduction technologies. Despite the high seroprevalence of B19V, there is a need for increased awareness, better reporting of TT-B19V cases, and stringent screening protocols to protect high-risk groups, including individuals with haematological disorders, immunodeficient patients, and pregnant women.

Mandatory B19V screening of donated blood, especially in high-demand regions, and retrospective evaluation of recipients with unexplained anaemia or clinical symptoms post-transfusion are necessary. Emerging technologies and future perspectives, such as high-sensitivity NAT assays and potential vaccination programs, promise further improvements in screening efficacy and overall blood safety. We believe that universal and stringent screening protocols for B19V in blood donors are vital in an era of increasing organ transplantations. The rise in renal transplant recipients, driven by the global increase in type 2 diabetes mellitus, underscores the critical need for enhanced measures to prevent B19V infections. These screening protocols will protect vulnerable patient populations, including pregnant women, ensuring safer transfusion practices and improving patient outcomes globally. Identifying specific research gaps, such as long-term outcomes of B19V infection and the efficacy of new screening technologies, is crucial for guiding future research and public health strategies.
